# Exploring E-Vape Aerosol Penetration into Paranasal Sinuses: Insights from Patient-Specific Models

**DOI:** 10.3390/ph18020142

**Published:** 2025-01-22

**Authors:** Amr Seifelnasr, Farhad Zare, Xiuhua Si, Jinxiang Xi

**Affiliations:** 1 Department of Biomedical Engineering, University of Massachusetts, Lowell, MA 01854, USA; amr_seifelnasr@student.uml.edu; 2 Department of Mechanical Engineering, Shiraz University, Shiraz JGV7+RG5, Iran; farhad.zare32@gmail.com; 3 Department of Mechanical Engineering, California Baptist University, Riverside, CA 92504, USA; asi@calbaptist.edu

**Keywords:** paranasal sinuses, e-vape, VG-PG, rhinosinusitis, nasal allergies, nose-sinus

## Abstract

**Background:** Acute and chronic sinusitis significantly impact patients’ quality of life. Effective drug delivery to paranasal sinuses is crucial for treating these conditions. However, medications from conventional devices like nasal drops, sprays, and nebulized mists often fail to penetrate the small ostia and reach the sinuses. This study aims to assess the effectiveness of e-vape-generated aerosols entering and filling paranasal sinus cavities, particularly the maxillary sinus. **Methods:** The aerosol droplets were generated using an electronic vaporizer (e-vape) and were composed solely of vegetable glycerin (VG) and propylene glycol (PG). Patient-specific, transparent nose-sinus models, including one with post-uncinectomy surgery, were used to evaluate the effectiveness of these e-vape-generated VG-PG aerosols in entering the sinuses under unidirectional and bidirectional airflow conditions. Visualizations from various nasal model views and lighting conditions were recorded. Particle size distribution measurements of the e-vape aerosol were conducted using a laser diffraction particle size analyzer. **Results:** E-vape-generated VG-PG droplets effectively enter paranasal sinuses under specific administration conditions. E-vape aerosol droplet size measurements revealed a mean particle size ranging from 2.895 to 3.359 μm, with a median particle size (D50) averaging 2.963 μm. The speed of aerosol entering the paranasal sinuses is directly proportional to the ostia size; larger ostia result in faster sinus entry. A continuous moderate flow is necessary to gradually fill the paranasal sinus cavities. The aerosol entry into sinuses was observed at 2 L/min and decreased with increasing flow rate. The mechanisms of aerosol entry involve maintaining a positive pressure gradient across the ostial canal, a non-equilibrium transverse pressure distribution, and a two-way flow through the ostium. Gravitational forces and recirculation currents further enhance the deposition of e-vape aerosols. Comparative tests showed that traditional delivery devices exhibited limited penetration into paranasal sinuses. **Conclusions:** This study demonstrated that e-vape-generated aerosols could serve as a vehicle for delivering active pharmaceutical ingredients (APIs) directly to the paranasal sinuses, improving treatment outcomes.

## 1. Introduction

Sinusitis, also known as rhinosinusitis, involves inflammation of the mucosa lining the nose and paranasal sinuses. Chronic sinusitis is characterized by inflammation lasting for more than 12 weeks and can manifest as chronic sinusitis without nasal polyps, chronic sinusitis with nasal polyps, and allergic fungal rhinosinusitis [1,2,3]. One of the most common types of sinusitis is maxillary sinusitis, which significantly contributes to the overall incidence of sinus diseases [4,5,6,7]. Chronic rhinosinusitis is prevalent among all age groups and ranks as the fifth most common reason for antibiotic prescriptions [8,9,10]. The causes of sinusitis are varied, including viral, bacterial, or fungal infections, as well as allergic reactions. Factors such as anatomical abnormalities, deficiencies in the immune system, and environmental influences can also contribute to the development of sinusitis [11,12]. The inflammation typically starts with mucosa swelling and ostia blockage, leading to mucus build-up and subsequent bacterial growth and infection [9,13]. Patients with rhinosinusitis frequently suffer from mucus oversecretion, mucosa inflammation, airway blockages, and compromised sinus drainage. These conditions result in a variety of symptoms, including nasal congestion, headaches, cough, bad breath, a diminished sense of smell, sore throat, facial swelling, fever, and fatigue [14,15,16]. Chronic rhinosinusitis can lead to a decrease in gray matter volume and cognitive impairments [17,18]. Acute rhinosinusitis may escalate to brain infections and other neurological complications [19,20].

**Figure 1:**
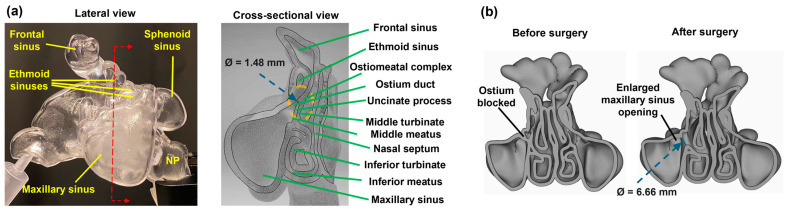
Nose-sinus terminology: (**a**) lateral and cross-sectional views of a patient-specific nose-sinus cavity model, depicting the maxillary, ethmoid, and frontal sinuses, uncinate process, ostiomeatal complex (highlighted by the orange-dashed region), and maxillary sinus ostia sizes (Ø); and (**b**) Cross-sectional back views of the model showing the left maxillary sinus ostium before and after uncinectomy surgery. NP: Nasopharynx.

The paranasal sinuses, which include the maxillary, frontal, ethmoid, and sphenoid sinuses (Figure 1a), are lined with respiratory epithelium consisting of goblet cells and ciliated cells. This lining is crucial for trapping and clearing pathogens and particles through mucociliary clearance. Chronic inflammation can alter this lining, leading to thickening of the basement membrane (i.e., subepithelial fibrosis) and an increase in goblet cells [10,13]. The paranasal sinuses perform several vital functions: they humidify and warm inhaled air, enhance voice resonance, provide protection against facial trauma, and reduce the weight of the skull [8,13]. These functions underscore the importance of maintaining sinus health and effectively treating sinus-related conditions. The maxillary sinus, the largest of the paranasal sinuses, is especially prone to various complications due to its anatomical structure, which predisposes it to obstructions, microbial colonization, viral infections, allergen exposure, fungal invasion, and chemical irritants [21]. Its relatively high ostial position exacerbates drainage issues. Additionally, the proximity to the frontal/ethmoidal sinuses and maxillary teeth makes it particularly prone for infection spread. Although paranasal sinus cancers are rare, they most commonly occur in the maxillary sinus [22].

Current treatments for sinusitis depend on the severity and duration of the condition. Standard treatments include antibiotics for bacterial infections, corticosteroids to reduce inflammation, and decongestants to alleviate nasal congestion. For severe cases, functional endoscopic sinus surgery (FESS) may be performed to open blocked sinuses and improve drainage when medication therapy fails (Figure 1b). Additionally, immunotherapy may be recommended for patients with significant allergic components. Other management strategies involve using nasal steroids with or without nasal saline irrigation, antihistamines, decongestants, and sometimes oral steroids [13,23,24,25,26]. Saline irrigation effectively clears foreign substances from the mucus layer, increases hydration of the deeper aqueous layer, restores ciliary beat frequency, and reduces local inflammatory mediators [27,28]. This approach is particularly helpful during viral respiratory infections, which can cause mucociliary dysfunction and mucus retention due to inflammation [29,30]. However, concerns exist that nasal irrigation might increase viral shedding and transmission [31]. Additionally, hypertonic saline can cause adverse local effects such as nasal burning and rhinorrhea [32,33]. The nasal cycle, which affects nasal geometry and resistance, can also influence irrigation effectiveness [34]. Salati et al. [35] found through physiology-based modelling that the head position and inflow direction significantly affected maxillary sinus volume filling. Inthavong et al. [36] conducted a numerical study using a squeeze bottle at a 45-degree forward head-tilt position, predicting moderate coverage (40%) of the ipsilateral maxillary sinus.

Effectively delivering medications to paranasal sinuses poses significant challenges due to the anatomical and physiological characteristics of the nasal passages and sinuses. The primary anatomical obstacles include the narrow nasal valve, the constricted entrance to the middle meatus, and the small ostia. The nasal valve’s minimal cross-sectional area restricts air flow and increases particle deposition due to inertial impaction [37,38]. The narrow entrance of the middle meatus, formed by the proximity between the middle and inferior conchae, limits efficient airflow ventilation and particle transport. The ostium, measuring 1 to 5 mm in diameter, is susceptible to blockages and bacterial growth, leading to infections within the maxillary sinus. The ostiomeatal complex, a primary site for sinusitis, becomes a critical target for aerosolized drug delivery [39,40,41]. These narrow openings restrict medication entry, hindering medications from reaching the site of inflammation. Traditional methods such as nasal sprays and irrigations often fail to penetrate these small openings adequately, limiting their effectiveness. A delivery protocol has been proposed to overcome the challenges posed by traditional methods, leveraging the gravity to deliver liquid drops to the ostiomeatal complex (OMC) and maxillary sinus [42].

Inhalation therapy presents several advantages, including quick action onset, targeted medication delivery, and reduced systemic side effects compared to oral methods [43,44,45,46]. However, the efficacy of inhalation therapy is modest, often resulting in an increased number of sinus surgeries [47,48,49,50]. Normal breathing does not sufficiently ventilate the middle meatus and sinuses, restricting sinus dosages. Consequently, most intranasal medications, like nasal sprays, are filtered by the nasal valve and do not reach the sinuses [51,52,53]. Efforts to improve drug delivery include bidirectional delivery during oral exhalation, which aims to prevent pulmonary penetration; however, over 95% of drugs still deposit in unintended areas, leading to significant waste and potential side effects [54,55,56,57,58,59]. Techniques such as humming flow or pulsating aerosols have shown improved dosimetry but still deliver less than 5% of the administered medication to the sinuses, with substantial wastage [60,61,62,63,64,65,66,67,68,69,70]. Addressing these challenges is crucial for improving the effectiveness of treatments for sinusitis and ensuring that medications can reach the site of inflammation within the sinuses.

This study was motivated by a novel in vitro finding that e-vape aerosols composed of vegetable glycerin (VG) and propylene glycol (PG) successfully entered and filled the paranasal sinuses under specific administration parameters. VG and PG, classified by the FDA as ‘generally recognized as safe’ (GRAS) for oral consumption, are widely used as safe, non-toxic ingredients in food, pharmaceuticals, and personal care products [71,72]. Rodent toxicity studies have demonstrated that VG and PG by inhalation do not adversely affect the nasal passages at certain exposure concentrations. A 90-day study found no toxicologically relevant effects of PG/VG aerosols at concentrations up to 1.52 mg/L PG plus 1.89 mg/L VG in rats [73]. Another 13-week study reported minimal mucous cell hyperplasia in the rat nasal cavity at 5 mg/L PG exposure, suggesting that PG and VG are generally well-tolerated at certain concentrations [74]. Thus, e-vape aerosols could be effective vehicles for delivering active pharmaceutical ingredients (APIs) directly to the paranasal sinuses, thereby improving treatment outcomes for conditions such as sinusitis. Additionally, e-vape aerosol delivery can address the challenges posed by current delivery methods, such as nasal sprays, liquids, and mists, which often fail to effectively penetrate the small ostia and adequately reach the sinuses.

The objective of this study is to assess the effectiveness of e-vape-generated aerosols composed of a mixture of VG and PG in entering and filling paranasal sinuses, particularly the maxillary sinus, under specific administration conditions. Specific aims include:

1. Prepare an assortment of patient-specific nose-sinus models.
2. Perform a detailed analysis of the e-vape aerosol characteristics, including particle size distribution and dispersion profile.
3. Assess the effectiveness of e-vape-generated VG-PG aerosols entering the paranasal sinuses in different nose-sinus models.
4. Evaluate the effect of uncinectomy surgery on VG-PG aerosol entry into the maxillary sinuses.
5. Understand the mechanisms underlying aerosol entry into isolated cavities using a dual-bottle set-up.

## 2. Results

### 2.1. E-Vape-Generated Aerosol Properties

#### 2.1.1. Aerosol Size Distribution

**Figure 2:**
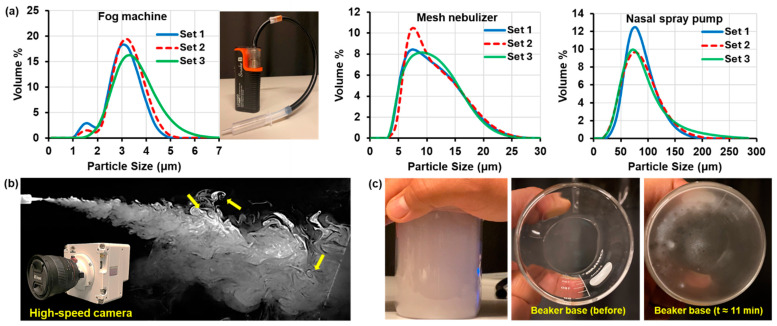
E-vape aerosol characterization: (**a**) particle size distribution profiles of aerosols dispensed from the mini fog machine, a mesh nebulizer filled with purified water, and a standard multi-dose nasal spray pump filled with saline solution; (**b**) high-speed imaging of the e-vape aerosol plume exiting the device nozzle in a still-air room, illustrating dense cloud formation and dispersion with gravitational sedimentation; (**c**) aerosol behavior in a sealed 300 mL beaker showing gradual dissipation and deposition, leaving a thin VG and PG oil mix layer on the bottom surface after 11 min. Yellow arrows denote mixing with the air.

The left panel of Figure 2a depicts the size distributions of aerosols dispensed from the custom-made aerosol dispensing device used in this study, alongside comparisons with a mesh nebulizer and a standard multi-dose nasal spray pump shown in the second and third panels. The mean particle sizes for the three measured sets were 2.895 μm, 3.097 μm, and 3.359 μm for the fog machine; 9.639 μm, 9.672 μm, and 9.940 μm for the mesh nebulizer; and 76.160 μm, 77.379 μm, and 79.146 μm for the multi-dose nasal spray pump. The peaks tilted to the left for the nebulizer and spray pump indicated more small-sized particles than large particles shown in the right tail (second and third panels, Figure 2a).

The mean of the median particle sizes (D50) from the three distribution curves in each plot was calculated. For the aerosol dispensing device using the mini fog machine, the mean D50 was 2.963 μm, compared to 8.387 μm for the mesh nebulizer and 69.411 μm for the multi-dose nasal spray pump. The aerosol dispensing device produced the smallest median particle size, while the multi-dose nasal spray pump generated the largest. Consistent with previous studies, e-cigarette aerosol particle sizes typically range between 1 and 5 μm [75,76,77], which aligns with the measurements in this study. These particles are larger than those from regular cigarette smoke, which are mostly in the submicron range (0.01–1 μm) [78].

#### 2.1.2. Mass Flow Rate

After operating the fog machine of the dispensing device five consecutive times, each for 15 s, the mass dispensed each time was measured as follows: 0.2050 g, 0.1801 g, 0.2195 g, 0.2313 g, and 0.1909 g. These measurements resulted in a mean net mass of dispensed aerosol of 0.2054 g, amounting to a mean mass flow rate of approximately 0.014 g/s (1.4 × 10^−5^ kg/s).

#### 2.1.3. Aerosol Dispersion Profile and Behavior in a Sealed Beaker

Figure 2b shows a high-speed snapshot acquired at 1000 fps of the aerosol plume exiting an electronic vaporizer into still air. The aerosol plume resembles a dense cloud characterized by vortices formed at the plume boundaries due to intensive mixing with the air (yellow arrows) and disperses with increasing distance from the nozzle. The micron-sized aerosol droplets are influenced by gravity, causing a gradual descent (Figure 2b).

To assess gravitational sedimentation, VG-PG aerosols were dispensed into a 300 mL beaker, filled, and sealed airtight for over 11 min. During this period, the dense aerosol cloud gradually dissipated, leaving a thin layer of residual VG-PG liquid at the bottom of the beaker (Figure 2c).

### 2.2. E-Vape Aerosol Entry into Paranasal Sinuses

#### 2.2.1. Optimal Flow Rate for Aerosol Entry and Filling in the Sinuses

**Figure 3:**
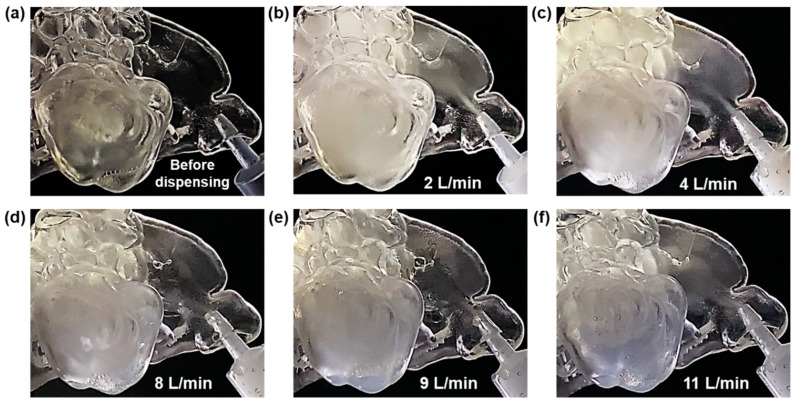
Side view of the maxillary sinus of F1-Right at various constant administration flow rates, showing the maximum aerosol filling observed (**a**) before dispensing, (**b**) at 2 L/min, (**c**) at 4 L/min, (**d**) at 8 L/min, (**e**) at 9 L/min, and (**f**) at 11 L/min. Each image depicts the maximum amount of aerosol that entered and filled the sinus, assessed based on visually observable concentration.

The optimal flow rate for maximizing aerosol entry and filling in the sinus cavities was assessed through visual inspection of aerosol concentration within the maxillary sinus of the F1-Right model (Figure 3).

The results showed that the effective flow rate for forced convective flow, facilitating aerosol delivery without escape through the administered nostril, ranged from 2 L/min to below 11 L/min (Figure 3b–f). The highest aerosol concentration within the sinuses was observed at 2 L/min (Figure 3b), decreasing with higher flow rates. At 11 L/min (Figure 3f) and beyond, there was no observable entry of aerosol into the maxillary sinus, likely due to reduced aerosol concentration near the maxillary sinus ostium in the ostiomeatal complex. Higher flow rates diluted the aerosol entering the sinus, increasing aerosol particle inertia, and reducing aerosol residence time, all decreasing the chance of a particle making a turn from the mean flow and entering the narrow ostium. While the speed of aerosol delivery increased with higher flow rates, the aerosol density significantly decreased as the flow rate approached 11 L/min (Figure 3c–f). The highest concentration and effective entry were observed at 2 L/min (Figure 3b), indicating that this is the optimal flow rate for aerosol delivery in the tested models. Therefore, a flow rate of 2 L/min was used for all subsequent measurements.

#### 2.2.2. Unidirectional Delivery

**Figure 4:**
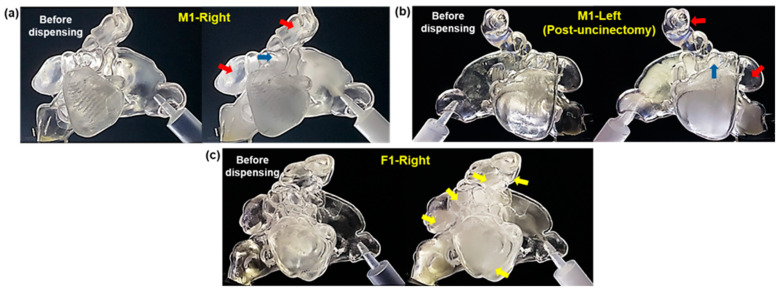
Images before and after aerosol dispensing under unidirectional inhalation conditions, demonstrating aerosol entry into paranasal sinuses of the M1 and F1 models: (**a**) M1-Right, (**b**) M1-Left after uncinectomy surgery, and (**c**) F1-Right.

Results of aerosol dispensing under unidirectional inhalation conditions demonstrated successful entry of aerosol into the paranasal sinuses in both the half- and complete cast models. E-vape aerosols were continuously dispensed until the sinuses were filled to their maximum holding capacity (i.e., with no further observable filling despite continued dispensing). Figure 4 shows images before and after dispensing to the M1-Right, M1-Left post-uncinectomy, and F1-Right half-models. The right and left maxillary sinuses of the M1 model (Figure 4a,b) were nearly filled, with some aerosol also entering a few ethmoid sinuses (blue arrows). However, due to ostia blockage to the frontal and sphenoid sinuses, these sinuses were void of any aerosol (red arrows). In contrast, the F1-Right model (Figure 4c) showed aerosol successfully entering and filling all paranasal sinuses (frontal, maxillary, ethmoid, and sphenoid), covering most cavities (yellow arrows).

**Figure 5:**
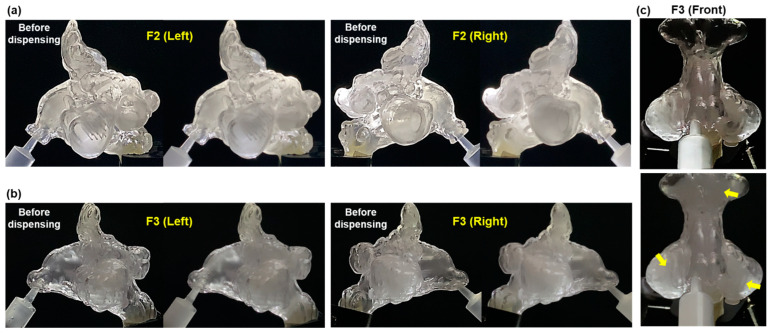
Various views of complete nasal models before and during aerosol dispensing show aerosol filling most of the paranasal sinus cavities: (**a**) F2, and (**b**) F3. (**c**) Front views of the F3 model highlighting the left and right maxillary sinuses as well as the frontal sinuses before (upper panel) and immediately after aerosol dispensing (lower panel). Yellow arrows point to aerosol in the sinuses.

Figure 5 depicts different views of the complete F2 and F3 nasal models before and after aerosol dispensing, highlighting the moments when the sinuses were filled. Similar to the M1-Right, M1-Left and F1-Right models, aerosol droplets successfully filled most paranasal sinus cavities. The front view of the F3 model (Figure 5c) reveals the majority of the left and right maxillary and frontal sinuses are filled with aerosol (yellow arrows).

**Figure 6:**
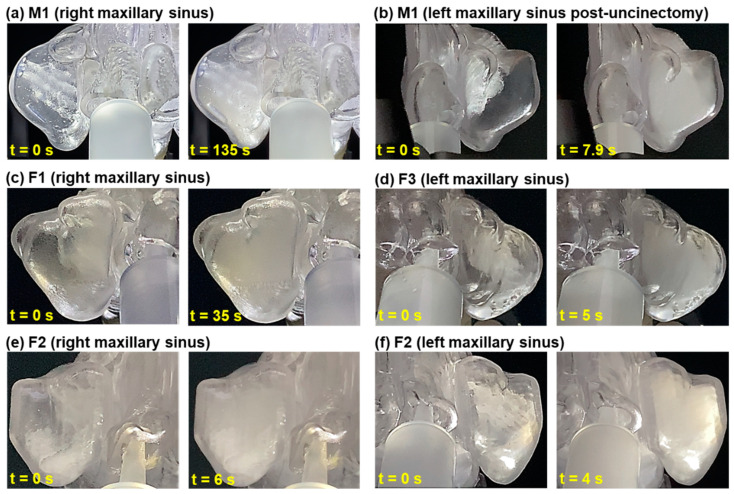
Front views of maxillary sinuses before and after aerosol administration for a length of time, t: (**a**) M1 (right side), (**b**) M1 (left side, post-uncinectomy), (**c**) F1 (right side), (**d**) F3 (left side), (**e**) F2 (right side), and (**f**) F2 (left side).

Figure 6 shows the front views of the right and left maxillary sinuses of the M1, F1, F2, and F3 models before and after aerosol administration for a length of time, t. The time required to fill the maxillary sinuses varied among models due to differences in ostia sizes, ranging from 4 to 135 s. The fastest filling time was recorded for the left maxillary sinus of the F2 model (Figure 6f), while the longest was for the right maxillary sinus of the M1 model (Figure 6a). In comparison, the left maxillary sinus of the M1 model (Figure 6b) filled much faster (7.9 s) due to the enlarged ostium resulting from uncinectomy surgery.

#### 2.2.3. Time Sequence of Aerosol Entry, Filling, and Post-Dispensing Dynamics in Paranasal Sinuses

**Figure 7:**
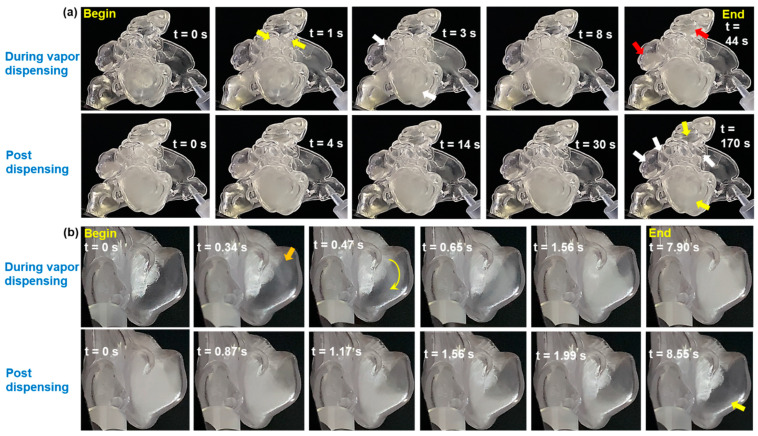
Time sequence of aerosol dynamics during and after aerosol dispensing within: (**a**) F1-Right, and (**b**) M1-Left post-uncinectomy surgery (front view); also see Supplementary Video S1.

Figure 7 depicts the time sequence of aerosol dynamics during and after aerosol dispensing within the right paranasal sinuses of the F1 model, and the left maxillary sinus of the M1 model post-uncinectomy surgery (front view), under constant unidirectional inhalation airflow.

During aerosol dispensing in the F1 model (first row, Figure 7a), aerosol began entering the maxillary sinus 1 s after initiation, descending from the ostium and partially filling the cavity. Aerosol also entered some anterior and posterior ethmoid sinuses (yellow arrows). Two seconds later, the maxillary sinus continued filling, and additional ethmoid sinuses started to receive aerosol (white arrows). By 8 s, the maxillary and ethmoid sinuses were fully saturated. Continuing dispensing beyond 8 s led to aerosol entry into the frontal and sphenoid sinuses, which gradually reached maximum capacity by 44 s (red arrows).

During the post-dispensing stage (second row, Figure 7a), the aerosol cloud within the paranasal sinus cavities dissipated in a slow and uneven manner. Some droplets escaped via the ostia, while others deposited, forming a thin liquid film lining the sinuses. The quantity of aerosol remaining and escaping depended primarily on ostia size and the degree of vacuum pressure at the ostium openings after dispensing stopped. Starting at 4 s post-dispensing, the aerosol that initially filled the sinuses gradually dissipated. By 170 s, most of the ethmoid sinuses and the sphenoid sinus had regained their initial transparency, indicating minimal or no deposition (white arrows). However, the opacity in the frontal and maxillary sinuses indicated significant deposition, with aerosol droplets forming a film across the inner surfaces (yellow arrows).

For the left maxillary sinus of the M1 model with an enlarged sinus opening post-uncinectomy surgery (first row, Figure 7b), aerosol entered the cavity through the ostium at the top within 0.34 s after dispensing began (orange arrow). The aerosol descended curvilinearly into the cavity (curved yellow arrow at t = 0.47 s), gradually dispersing and filling the sinus with significant recirculating currents generated in the process. At t = 7.9 s, the maxillary sinus was fully saturated, and additional dispensing beyond that point did not result in any noticeable increase in aerosol concentration. More details can be viewed in Supplementary Video S1.

During the post-dispensing stage (second row, Figure 7b), the much larger maxillary sinus ostium of the M1 model resulted in much quicker aerosol escape compared to the F1 model. At almost 2 s after stopping aerosol administration, most of the aerosol had escaped from the M1’s maxillary sinus via the enlarged ostium. However, the slight opacity in the bottom section of the sinus cavity (yellow arrow at t = 8.55 s) indicated that a small fraction of aerosols deposited and coated that section of the cavity’s surface.

#### 2.2.4. Bidirectional Delivery

**Figure 8:**
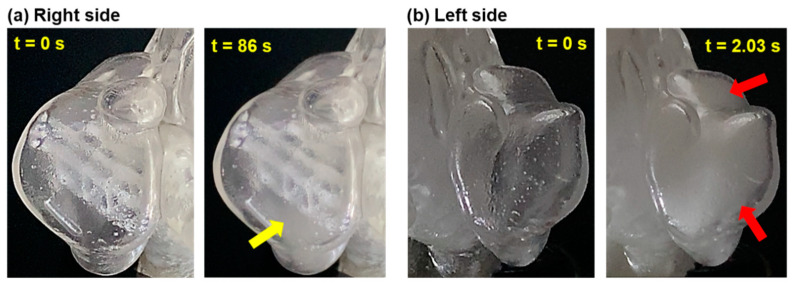
Front views of the maxillary sinuses of the M1 nasal model before and after a period of time following the initiation of aerosol administration using bidirectional delivery: (**a**) right maxillary sinus, and (**b**) left maxillary sinus (post-uncinectomy).

The bidirectional aerosol delivery tests showed effectiveness comparable to unidirectional delivery in facilitating aerosol entry into the sinuses. Figure 8 shows the front views of both the right maxillary sinus and the left maxillary sinus (post-uncinectomy) of the M1 nasal model before and after a period of aerosol administration. While the complete M1 model contained unavoidable artifacts (3D-printing support material) from the 3D-printing process that slightly obscured transparency, Nevertheless, aerosol dynamics within the cavities remained observable, though with reduced clarity compared to the half-models.

Using bidirectional delivery, the aerosol filled the right maxillary sinus after 86 s of continuous dispensing (yellow arrow, Figure 8a). By contrast, the aerosol filled the left maxillary sinus (Figure 8b), which had a surgically enlarged ostium, in just 2.03 s (red arrows).

#### 2.2.5. Aerosol Filling with Normal and Surgically Enlarged Ostia

**Figure 9:**
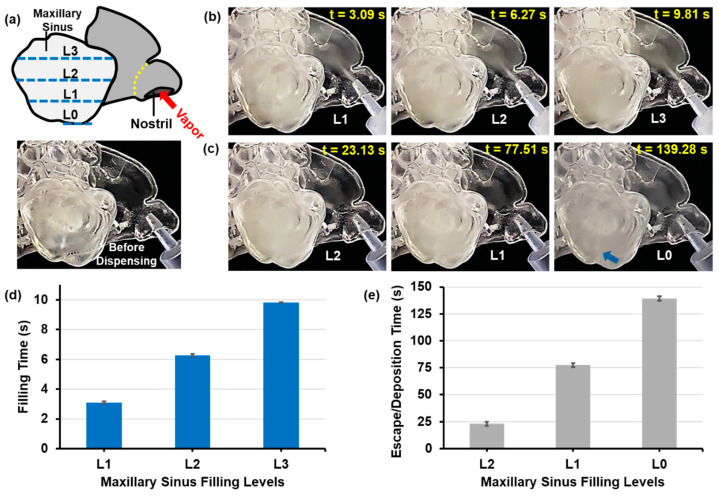
Aerosol filling and dissipating dynamics in the maxillary sinus of the F1-Right model (featuring a natural ostium): (**a**) schematic showing levels L0-L3, (**b**) sequence during aerosol dispensing, (**c**) sequence after cessation of aerosol dispensing, (**d**) times during aerosol dispensing, and (**e**) times after cessation of aerosol dispensing for the aerosol within the left maxillary sinus to reach predefined levels (L0–L3). The yellow dotted line denotes the nasal valve, and the blue dashed lines denote the aerosol filling/emptying levels. The blue arrow denotes the residual oily film. Dynamic sinus filling can be viewed in Supplementary Video S2.

**Figure 10:**
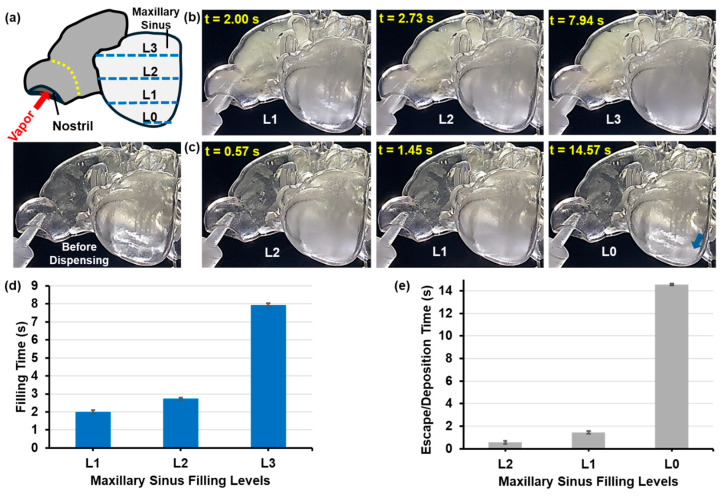
Aerosol filling and dissipating dynamics in the maxillary sinus in the M1-Left model (post-uncinectomy): (**a**) schematic showing levels L0–L3, (**b**) sequence during aerosol dispensing, (**c**) sequence after cessation of aerosol dispensing, (**d**) times during aerosol dispensing, and (**e**) times after cessation of aerosol dispensing for the aerosol within the left maxillary sinus to reach predefined levels (L0–L3). The yellow dotted line denotes the nasal valve, and the blue dashed lines denote the aerosol filling/emptying levels. The blue arrow denotes the residual oily film. Dynamic sinus filling can be viewed in Supplementary Video S3.

Figure 9 and Figure 10 show the times for aerosol within the right maxillary sinus of the F1 model and the left maxillary sinus of the M1 model (post-uncinectomy surgery) to reach predefined levels (L0–L3) during and after dispensing, as per the schematics in Figure 9a and Figure 10a. The tests were conducted at the optimal constant inhalation flow for aerosol delivery of 2 L/min. The right maxillary sinus of the F1 nasal model represents a case with a natural ostium, whereas the left maxillary sinus of the M1 model represents a case with a surgically enlarged ostium. Due to the challenges in identifying clear boundaries associated with the aerosol at the predefined levels during and after aerosol dispensing, times for the aerosol to pass the levels were measured multiple times to account for slight subjectivity in assessing these boundaries. The time taken to reach the predefined levels (L0–L3) from one level to another changed in a non-linear fashion. The transient sinus filling can be viewed in Supplementary Video S2.

For the F1-Right model, the mean times to reach L1, L2, and L3 during aerosol administration phase were 3.09 s, 6.27 s, and 9.81 s, respectively (Figure 9b,d). After aerosol administration ended, the time for the aerosol within the sinus cavity to decrease to levels L2, L1, and L0 were substantially longer: 23.13 s, 77.51 s, and 139.28 s, respectively (Figure 9c,e). Meanwhile, the concentration of the aerosol decreased progressively due to escaping via the ostium or depositing on the sinus walls. The opacity of the maxillary sinus after aerosol dispensing (blue arrow, Figure 9c) is due to the residual oily film lining the inner sinus walls after gradual aerosol deposition.

Compared to the F1-Right model, the much-enlarged opening to the maxillary sinus in the M1-Left model after uncinectomy surgery resulted in significantly different aerosol dynamics during and after aerosol administration. Also, see Supplementary Video S3. From the moment the aerosol exited the dispensing nozzle and entered the left nostril, it took 2 s to reach L1 and an additional 0.73 s to reach L2 (Figure 10b,d). However, an average of 7.94 s was needed for the aerosol to reach the L3, demonstrating the non-linear aspect of the time required to fill specific levels of the sinus cavity.

The time for the aerosol to pass the levels during the post-dispensing phase also varied significantly between the M1-Left and F1-Right models (Figure 9c,e vs. Figure 10c,e). Due to the significantly larger ostium opening, the aerosol escaped the M1-left’s sinus cavity at a much higher rate, decreasing from L3 to L2 in 0.57 s and to L1 in a mean time of 1.45 s. For the aerosol to be visually clear from the sinus, a mean time of 14.57 s passed after aerosol dispensing stopped (Figure 9e). As with the F1-Right model, a degree of opaqueness of the transparent cavity wall resulted at the end of the post-dispensing phase due to the presence of residual oil film (blue arrow, Figure 10c). However, it was less pronounced compared to the F1-Right model, as much less aerosol remained in the cavity due to the larger ostium, resulting in less deposition on the inner walls of the maxillary sinus cavity, mostly toward the inferior cavity via gravitational sedimentation (Figure 9c vs. Figure 10c).

### 2.3. Mechanisms of E-Vape Aerosol Entry and Deposition into the Paranasal Sinuses

**Figure 11:**
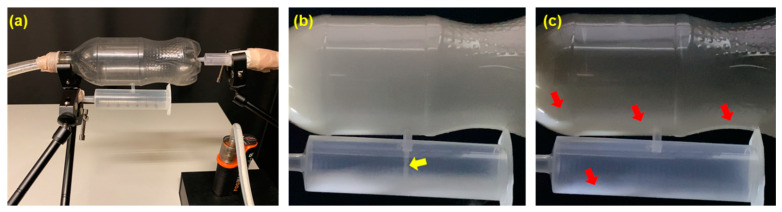
Dual-bottle setup mimicking the nose-sinus cavities: (**a**) Experimental setup used to study the mechanism of aerosol entry and deposition within a sinus cavity. (**b**) Aerosol entry into the cavity due to a positive pressure gradient (yellow arrow). (**c**) Residual oil film across the surface lining of the bottle and syringe post-dispensing of aerosol, indicated by red arrows.

The experimental setup described in Section 2.4 was utilized to study the mechanism of aerosol entry and deposition within the paranasal sinuses (Figure 11a). The paranasal sinuses are dead-end cavities, each typically featuring a single, narrow primary ostium that allows fluid to flow both into and out of the sinus. For fluids to enter the sinus through the ostium, they must make a 90° bend. Additionally, the particles within the flowing fluid must be of a suitable size to effectively penetrate the small ostia. Particles that are too large are likely to deposit via inertial impaction and struggle to follow the abrupt turn into the small ostia openings.

Based on observations, it was deduced that the primary mechanism driving aerosol entry was a pressure gradient (ΔP) generated across the opening of the syringe within a specific range of flow rates. This pressure gradient is described by the equation ∆P = P_Nasal_ − P_Sinus_. The flow rate (Q) of the aerosol entering the sinus cavity is governed by the relationship between the pressure gradient and the resistance (R) of the pathway, expressed as Q = ∆P/R.

Within a specific range of valve openings (i.e., flow rates), and using constant flow, the aerosol successfully entered the syringe (yellow arrow, Figure 11b). However, the higher the flow rate, the less dense the aerosol flowing through the bottle became, resulting in a lower concentration of aerosol entering and filling the syringe cavity. When the vacuum pressure at the outlet reached a certain high limit, the aerosol flowing through the bottle became so diluted that no aerosol was observed entering the cavity of the syringe body. Conversely, if the flow rate was too low, it resulted in backflow and aerosol leakage out of the bottle via the inlet, preventing effective entry into the sinus (syringe body) cavity. A vacuum pressure corresponding to a flow rate at the outlet end of around 2 L/min resulted in the highest flow of aerosol into the syringe. At this flow rate, the aerosol entering the sinus smoothly descended to the bottom of the syringe cavity, gradually filling it.

Thus, maintaining a specific flow rate range that allows for sufficient concentration (density) of administered aerosol flowing into the bottle (i.e., nasal passage cavity) as well as a sufficient pressure gradient across the opening to the syringe (i.e., sinus cavity) is crucial for aerosol entry into the sinus cavity. This mechanism underscores the importance of pressure-driven flow for effective e-vape aerosol delivery to the sinuses. Moreover, the effective diameter and length of the ostium play a significant role in facilitating or impeding aerosol entry. The sinus cavity must also accommodate two-way traffic, wherein the air inside the cavity is displaced by the entering aerosol. Once inside, the aerosol that has not escaped post-dispensing and remains within the sinus cavity dissipates by gradually depositing and returning to the liquid state, leaving a layer of oil on the surface. The presence of a residual oil film across the surface lining of the bottle and syringe (red arrows, Figure 11c), observed after being filled with e-vape aerosol, supports this conclusion.

**Figure 12:**
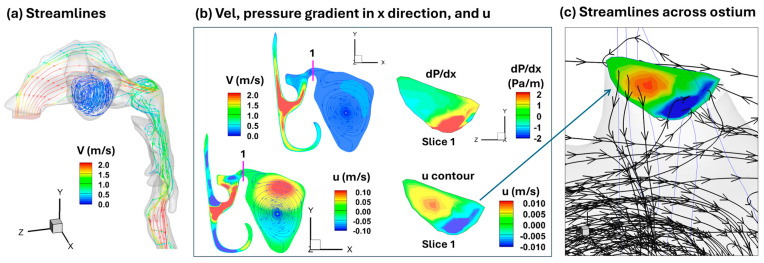
Numerical simulations: (**a**) streamlines, (**b**) velocity and pressure gradient, and (**c**) non-equilibrium pressure distribution in transverse cross-section, showing two-way traffic flow.

To further explore the mechanisms underlying the e-vape aerosol penetration into paranasal sinuses, numerical simulations of the inspiratory airflow in the nose-sinus model were conducted, as shown in Figure 12. There are indeed airflows entering the sinus, but at a very low flow speed, as illustrated by the blue color of the streamlines inside the sinus. Figure 12b shows the airflow and pressure dynamic within the ostium connecting the middle meatus and maxillary sinus cavity, with a non-equilibrium pressure and velocity distribution, with both positive and negative pressure gradients (dP/dx), as well as positive and negative axial velocities (u), occurring across the same transverse cross-section (Figure 12b, right panels). Figure 12c shows clearly that airflow streamlines go across the ostium in both directions (i.e., two-way flow traffic), allowing aerosols in the middle meatus enter the maxillary sinus constantly.

### 2.4. Low Sinus Entry from Nasal Sprays and Mists (Nasal Spray Pump, Squeeze Bottle, and Nebulizer)

**Figure 13:**
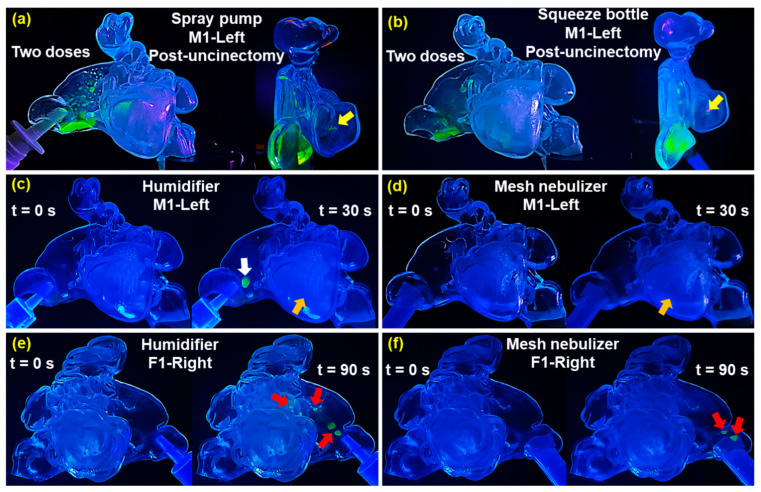
Deposition results using alternative delivery devices. M1-Left (post-uncinectomy): (**a**) multi-dose nasal spray pump, (**b**) squeeze bottle, (**c**) humidifier, and (**d**) mesh nebulizer. F1-Right (maxillary sinus natural ostium): (**e**) humidifier, (**f**) mesh nebulizer.

Under the same administration factors used in the aerosol delivery tests, namely an upright head position, an inhalation flow rate of 2 L/min, and an administration angle of around 45° from the horizontal pointing towards the middle meatus, tests with alternative delivery devices showed either insignificant or no noticeable deposition in most paranasal sinuses. Figure 13 shows the results before and after administration. As seen in Figure 13a, using the M1-Left model (post-uncinectomy), two applications of the nasal spray pump (averaging 95 mg per application) resulted in spray deposition primarily in the nasal vestibule and anterior nasal cavity, mainly along the floor, with some scattered liquid droplets and film in the superior and middle respiratory region and within the middle meatus. Insignificant liquid deposition was observed in the maxillary sinus (yellow arrow), and no deposition was observed in the remaining paranasal sinuses.

Using the squeeze bottle saline nasal moisturizing spray (Figure 13b), two applications led to deposition in the nasal vestibule and the superior and middle nasal cavities. Liquid film was also observed within the middle meatus, most of which translocated and settled on the nasal cavity floor. Very insignificant deposition occurred in the maxillary sinus (yellow arrow), with no deposition observed in the frontal, ethmoid, or sphenoid sinuses.

The administration of purified water mist into the M1-Left model (post-uncinectomy) using the humidifier (Figure 13c) and the mesh nebulizer (Figure 13d) did not result in observable deposition within the frontal, ethmoid, or sphenoid sinuses. However, a faint mist cloud was seen in the maxillary sinus after 30 s of continuous dispensing with both devices (orange arrows, Figure 13c,d). The surgically enlarged maxillary sinus ostium facilitated the mist entry into the maxillary sinus. With the humidifier, a water film developed just beyond the nasal valve after continued dispensing (white arrow, Figure 13c).

In contrast, the F1-Right model with a natural maxillary sinus ostium showed no deposition of liquid water droplets in the paranasal sinuses after 1 min of dispensing from the mesh nebulizer or humidifier. Scattered water droplets were observed in the anterior turbinate region downstream of the nasal valve (red arrows, Figure 13e,f), indicating elevated water deposition in that area.

## 3. Discussion

This study demonstrated that e-vape-generated aerosols from a mixture of vegetable glycerin (VG) and propylene glycol (PG) could effectively enter the maxillary sinus under controlled administration conditions. This successful entry highlights the potential of e-vape-generated aerosols as a promising vehicle for delivering e-vape based delivery systems.

### 3.1. Factors Contributing to Effective Entry and Deposition of E-Vape Aerosol into Sinuses

The study identified several factors contributing to the effective entry of e-vape aerosols into the maxillary sinus. Effective entry is primarily driven by maintaining a positive pressure gradient across the sinus ostial canal, critical for overcoming the anatomical barriers posed by this small and often narrow conduit. Additionally, for the aerosol to fill the cavity, it must displace the air within. Since there is only one ostium, the displaced air exits through the same opening, resulting in a two-way flow of aerosol into the sinus and air out of the sinus. This continues until the sinus reaches its maximum holding capacity under prescribed aerosol administration flow conditions. Moreover, this two-way flow through the small ostium creates a non-equilibrium pressure distribution across any axial cross-section of the ostium, as shown in the complementary numerical analysis (Figure 12a–c). Particularly, in Figure 12c, airflow streamlines traverse the ostium in both directions, enabling aerosols present in the middle meatus to continuously enter the maxillary sinus. This mechanism is different from that of sinus delivery using pulsating flows, where resonance facilitates aerosol entry into the sinus [69].

Tests to assess the optimal flow rate for preventing aerosol escape and generating an effective pressure gradient showed that a range of 2 L/min to below 11 L/min was effective (Figure 3). However, higher flow rates diluted the administered aerosol, resulting in a lower concentration and quantity of aerosol entering and filling the sinus. The highest concentration and most effective aerosol entry were observed at a flow rate of 2 L/min (Figure 3b), identifying this as the optimal flow rate for aerosol delivery in the tested models. At this flow rate, gravity was more dominant than convection within the maxillary sinus cavity of the F1-Right model. With an upright head orientation, aerosol droplets entering the ostium flowed smoothly downward, filling the sinus from the bottom up until it reached the ostium opening. At higher flow rates, the aerosol entering the sinus became less dense, and convection became more dominant than gravity due to increased flow velocity. This caused turbulent recirculation and dispersion within the cavity, highlighting the importance of controlling aerosol flow conditions.

The diameter and length of the ostium play a significant role in facilitating aerosol entry, with larger and shorter ostia allowing more efficient aerosol penetration, consistent with previous studies [69,70]. The speed of aerosol delivery to the paranasal sinuses was found to be directly proportional to ostia size: larger ostia resulted in faster delivery, while smaller ostia slowed the process. The duration required to achieve complete or substantial filling of all paranasal sinus depends on the size of the smallest ostium within the nasal cavity. This was demonstrated in the tests using the F1-Right nasal model (Figure 7a and Figure 9b), where less than 10 s of continuous aerosol dispensing was needed to fill the maxillary sinus and most ethmoid sinuses, while an additional 34 s was required to fill the frontal and sphenoid sinuses to their maximum aerosol-holding capacity.

According to Abouali et al. [79], endoscopic surgery significantly enhanced maxillary sinus ventilation, accelerating particle entry into these sinuses. Similarly, Amjadimanesh et al. [43] underscored the uncinate process’s vital role as a barrier preventing microparticles from entering the sinuses. Their research demonstrated that uncinectomy surgery alone promoted the easy entry and deposition of microparticles on sinus walls. Additionally, their findings suggest that combining an uncinectomy with a middle meatal antrostomy (MMA) markedly increases particle deposition within the maxillary sinus. This study has indeed demonstrated that uncinectomy surgery significantly enhances aerosol entry and deposition. The results of the tests using the F1-Right model, featuring a natural ostium, and the M1-Left model with a significantly enlarged maxillary sinus ostium after uncinectomy surgery (with an opening size of 6.66 mm, compared to 1.94 mm in the F1-Right model, as shown in Figure 1a,b), as illustrated in Figure 9 and Figure 10, support this conclusion.

For the F1-Right model, the times to reach levels L1, L2, and L3 during aerosol administration were 3.09 s, 6.27 s, and 9.81 s, respectively. Post-dispensing, the times for aerosol droplets to escape or deposit were 23.13 s, 77.51 s, and 139.28 s. The residual oil film in the sinus cavity indicated substantial deposition. In contrast, the M1 model with a surgically enlarged ostium showed faster aerosol entry, reaching L1, L2, and L3 in 2 s, 0.73 s, and 7.94 s, respectively. However, the larger ostium facilitated quicker aerosol escape post-dispensing, with times from L3 to L2, from L2 to L1, and for complete clearance being 0.57 s, 1.45 s, and 14.57 s. Less aerosol deposition was observed due to rapid aerosol escape. These findings highlight that a natural ostium results in slower aerosol entry and prolonged retention, whereas an enlarged ostium allows for faster entry and quicker clearance, affecting the overall deposition of e-vape aerosols within the sinus cavities.

Particle size plays a crucial role in aerosol entry and deposition. Measurements showed that the aerosols generated from a 50/50 VG-PG formulation by the custom aerosol dispensing device used in this study had a median particle size (D50) averaging 2.963 μm, smaller than those from a mesh nebulizer, and much smaller than those from a nasal spray pump (Figure 2). Similar results were also reported by Bruneau et al., who measured smaller particle sizes of beclomethasone dipropionate from a vaping drug delivery system than nebulization [80]. The ability of the e-vape aerosol to penetrate the small ostia of the sinus cavities is highly dependent on maintaining this particle size range. Particles that are too large are prone to deposit via inertial impaction and struggle to follow the abrupt turn into the small ostia openings. By contrast, standard nasal devices, including a multi-dose nasal spray pump, a squeeze bottle saline nasal spray, a mesh nebulizer, and a humidifier, failed to deliver adequate aerosolized medications to the sinuses under the same experimental conditions (Figure 13).

### 3.2. Practical Implications of the Study

The findings from this study suggest that e-vape aerosols, composed of a 50/50 mix of VG and PG, have the potential to serve as an effective medium for delivering active pharmaceutical ingredients (APIs) directly to the paranasal sinuses. The ability of e-vape aerosols to penetrate the small ostia and fill the sinus cavities could provide a more effective alternative to traditional nasal sprays, drops, and mists, which often fail to adequately reach the sinus cavities. Moreover, if proven safe, this method could reduce the need for invasive surgical procedures like uncinectomy or maxillary antrostomy, currently performed to enlarge the sinus ostia and improve drug delivery.

The study underscores the importance of optimizing e-vape aerosol delivery parameters to ensure effective treatment. Factors such as particle size, flow rate, and administration angle must be carefully controlled to maximize aerosol entry and deposition within the sinuses. This optimization could pave the way for the use of e-vape aerosols in clinical settings, provided that its safety and efficacy are thoroughly evaluated in future studies. Moreover, bidirectional delivery was identified as a practical approach for administering e-vape aerosols to the sinuses (Figure 8). This method, which involves sealing the nasopharynx to prevent pulmonary delivery, demonstrated effectiveness in the experimental tests and can be incorporated into a delivery device/system. The bidirectional delivery approach directs aerosol droplets toward the paranasal sinuses, enhancing the likelihood of effective deposition and treatment. However, an important consideration for future development is the potential impact of heating the PG-VG mixture on the integrity of active pharmaceutical ingredients (APIs) and their effective transfer into the aerosol. High-power devices, such as the one used in this study (40 W), generate significant heat, which may degrade thermally sensitive drugs or alter their pharmacological properties. Additionally, the transfer efficiency of APIs into the aerosolized phase warrants investigation to ensure precise dosing. Note that the e-vape power can also notably affect the aerosol count and size distribution, as demonstrated by Floyd et al. [75]. These factors, though beyond the scope of this study, are critical for the clinical translation of this technology and should be addressed in future studies to optimize dosing strategies and ensure drug stability under thermal stress.

Additionally, the study provides insights into how active and passive e-vape smoking influence aerosol penetration into the paranasal sinuses. Understanding aerosol behavior and deposition within the sinuses can inform public health policies and smoking regulations, particularly in indoor environments where passive exposure to e-vapor could occur. The results highlight the importance of considering both the particle size and the administration parameters when evaluating the potential health impacts of e-vape smoking exposure. Understanding the deposition patterns of e-vape aerosols can help in developing strategies to mitigate their effects on individuals with respiratory conditions or allergies, emphasizing the need for controlled indoor air quality and effective filtration systems. Considering that the time required to fill the sinus is generally much longer than a breathing cycle (5 s) and that a positive pressure gradient is needed for aerosol entry, the bidirectional delivery method is recommended. This method dispenses aerosols into one nostril and adds a resistor to the other nostril, forming a continuous U-shaped flow with a controlled flow rate. Another benefit of this method is that it is independent of breathing maneuvers, and thus will be well suited for administration with long durations.

### 3.3. Limitations

While the study presents promising results of aerosol entry into sinuses, several limitations must be acknowledged. The anatomically accurate nasal models used in this study were crucial for evaluating aerosol delivery to the paranasal sinuses. However, they represent a limited range of nasal geometries. Including a broader variety of patient-specific nasal models would enhance understanding of how structural variability affects aerosol distribution, thus enriching assessments of aerosol delivery effectiveness across diverse populations. Furthermore, the in vitro experiments were conducted using 3D-printed nasal models. The SLA resin used to print the nasal models does not accurately replicate the characteristics of actual nasal airway tissue or the mucosal tissue lining the paranasal sinuses. A thorough analysis of the interactions between the e-vape aerosols and the nasal mucosa compared to those with the resin surface is essential to assess how accurately the models mimic real nasal cavity conditions and to understand the implications for the study’s findings.

In this study, the quantification of e-vape aerosol delivery to the sinuses remained largely qualitative, even though aerosol filling/emptying durations were measured in several models. Direct in vivo measurements are yet infeasible due to the inaccessibility of paranasal sinuses to measurement instruments. In experimental studies like this one, the anatomical continuity between the nasal passage and sinuses makes it challenging to differentiate dosages between these two regions. Additional challenges arise when sinus and nasal casts are prepared separately and joined for testing. First, any leakage at the connection interface can significantly affect aerosol entry into the sinuses. Second, when using tape to seal these interfaces, residual adhesive can introduce dosing uncertainties, which is particularly significant given the potentially minute quantities of aerosol entering the sinuses. Third, the small diameter of the ostium complicates rinsing procedures. One promising approach for dose quantification is colorimetry. This method involves directly dispensing known doses into sinus-only casts via the ostium while recording color variations. These recordings can then serve as references for estimating aerosol doses in the sinuses during in vitro tests through color-matching analysis.

Additionally, the presence of unavoidable 3D-printing support material and artefacts within some of the models from the 3D printing process may have influenced aerosol dynamics and deposition patterns (Figure 8). Visualization of aerosol within the sinus cavities was challenging due to its low density in some regions and glare from the model walls, potentially impacting observation accuracy. Moreover, while VG and PG are recognized as safe and non-toxic by the FDA [71,72], their effects in aerosolized form on mucosal health and mucociliary function need further evaluation. Clinical studies are required to validate the nonclinical efficacy demonstrated in this study and to ensure the safety and effectiveness of e-vape aerosol delivery for treating sinusitis and nasal allergies. Moreover, this study mainly provided proof of concept for e-vape aerosol penetration into the paranasal sinuses. Further research is necessary to develop e-vape-based medical devices or drug delivery protocols for sinus-targeted therapeutic applications.

## 4. Materials and Methods

### 4.1. Study Design

An aerosol dispensing device was custom-made to explore the effectiveness of e-vape aerosols entering the paranasal sinuses via the small ostia and the extent to which the aerosol can fill them up. This device comprised an electronic vaporizer, or e-vape (LENSGO Smoke B, Shenzhen, Guangdong, China), a 10 mm inner diameter silicone tube, and an administration nozzle constructed from the barrel and nozzle of a 12 mL disposable plastic dental irrigation syringe (Protector). The liquid used in the fog machine consisted of a 50/50 mix of vegetable glycerin (VG) and propylene glycol (PG). Note that VG is not a single component, containing 95–99% 1,2,3-propanetriol (C_3_H_8_O_3_), as well as trace amounts of water, fatty acids, and minerals such as sodium, potassium, and chloride [80]. The e-vape dispensed aerosol droplets at a rate of 0.6 L/min, measured using a hot-wire anemometer (Testo 405i, Titisee-Neustadt, Germany). The curved end of the syringe was trimmed off to create a tapered nozzle with a length of 17 mm and a circular orifice with a diameter of 3 mm. The silicone tube was connected to the outlet of the mini fog machine at one end and to the base of the syringe at the other end.

**Figure 14:**
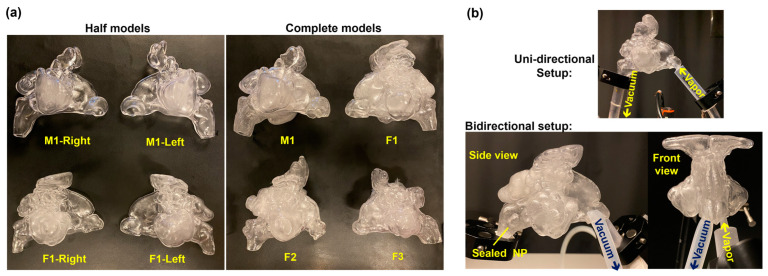
Nose-sinus casts along with experimental setups: (**a**) half- and complete models, (**b**) unidirectional and bidirectional setups. M1: male model 1; F1–3: female models 1–3.

Nine patient-specific nose-sinus casts were utilized, 3D-printed using a transparent resin (Figure 14a). These anatomically accurate models, featuring either one passage (half-model) or two passages (complete model), provided a detailed representation of the nose-sinus cavities, allowing for precise observation and assessment of aerosol distribution.

In vitro tests involved two distinct experimental setups: one employed unidirectional airflow, while the other utilized bidirectional flow (Figure 14b). The rationale for using bidirectional delivery stemmed from a practical perspective, aiming for the potential future development of an intranasal delivery device. This device would administer e-vape aerosol using a bidirectional method, sealing the soft palate during aerosol dispensing, thus preventing delivery to the lungs—an outcome not feasible with unidirectional delivery. Unidirectional flow was used to demonstrate the method’s effectiveness, with which aerosols can enter the sinuses under controlled conditions. Bidirectional flow was employed to show that, even with different flow conditions, the method would still work given specific delivery parameters.

In all experimental runs, whether using unidirectional or bidirectional flows, the models were oriented in an upright head position, with the nasal cavity floor levelled horizontally. The e-vape nozzle was inserted into the nostril with its tip at the entrance to the internal nasal valve of each model, allowing direct aerosol entry into the nasal passage. The nozzle was pointed towards the middle meatus at an angle of around 45° from the horizontal and placed in the nostril corresponding to the passage housing the targeted sinuses.

In the unidirectional setup, the nasopharynx was connected via a flexible tube to a vacuum (Robinair 3 CFM, Warren, MI, USA) to generate a constant inhalation flow. Tests were conducted to determine the optimal flow rate for maximizing aerosol entry and filling in the paranasal sinuses, particularly the maxillary sinus, based on visual observations of aerosol concentration in the nose-sinus cast models used in this study. The tests focused on aerosol dynamics within the maxillary sinus of the F1-Right nose-sinus model, which has a natural ostium. The optimal flow rate was defined as the rate that prevented aerosol droplets from escaping from the administered nostril while creating a pressure gradient across the ostium, maximizing aerosol entry and sinus filling. This focus on the maxillary sinus is particularly important, as maxillary sinusitis is one of the most common types of sinusitis and significantly contributes to the overall incidence of sinus diseases [4,5,6,7]. The flow rate was regulated via a flow meter (Omega, FL-510, Stamford, CT, USA). Tested flow rates included 2 L/min, representing gentle inhalation; 4 L/min, representing moderate inhalation; and 8 L/min, 9 L/min, and 11 L/min, representing various degrees of strong inhalation. Tests were conducted using unidirectional delivery with both the half- and complete nose-sinus models. For the bidirectional setup, only complete models were utilized. To simulate bidirectional delivery, the nasopharynx was sealed off to mimic soft palate sealing during delivery, and the vacuum was connected via a flexible silicone tube to the nostril contralateral to the one into which the e-vape aerosols were administered.

A 12 megapixel high-resolution camera captured the process during aerosol dispensing from the side and front views of the models. A high-intensity flashlight (Mankerlight MK38, Dongguan, Guangdong, China) was used to illuminate the background and placed in a location that would maximize the contrast to distinguish the dispensed aerosol from the rest of the model. Recorded videos were analyzed to assess the effectiveness of the filling of the paranasal sinuses with e-vape aerosols, the distribution patterns of the aerosols within the sinus cavities, as well as the time required to fill them.

### 4.2. Nose-Sinus Models

To account for intersubject variability in nasal passage morphology, a set of nasal cavity models, including their associated paranasal sinuses, were used in the in vitro tests (Figure 14a). The models were developed from CT scans of one adult male and three adult females. The usage of CT images was approved by the University of Massachusetts Lowell institutional review board. A total of nine cast models were used: four half-models representing the right and left nasal passages and sinuses of one male (M1) and one female (F1), and five complete models associated with the adult male (M1) and three females (F1–3). The half-models allowed inspection of aerosol dynamics from both the outer (turbinate) and inner (septum) directions, avoiding visual obstruction from the other passage. The complete models included two for the adult male (M1), representing the anatomical structures before and after uncinectomy surgery performed on the patient’s left side. The post-surgery model was used to assess the effect of a surgically enlarged ostium on the speed of aerosol entry into the maxillary sinus.

Figure 1a shows a front coronal sectional view of the right nose-sinus cavity of the M1 and F1 models, highlighting some of the paranasal sinuses, uncinate process, ostiomeatal complex region (orange-dashed area), and the maxillary sinus ostia with their opening sizes (Ø). The ostia on the right side of both the M1 and F1 nasal models represent natural ostia. Conversely, the left maxillary sinus ostium in the right image of Figure 1b (showing back coronal sectional views of the M1 nasal cavity before and after uncinectomy) is surgically enlarged, resulting in a much wider opening to the left maxillary sinus. (Ø = 6.66 mm compared to 1.48 mm in M1-Right).

A Stratasys Neo450s 3D printer (Eden Prairie, MN, USA) was used to produce the nasal casts using a clear, rigid stereolithography (SLA) resin (Somos^®^ WaterShed XC 11122, Stratasys, Eden Prairie, MN, USA), resulting in transparent hollow casts. Utilizing transparent casts provides a significant benefit by allowing real-time observation of e-vape aerosol dynamics and distribution within the nasal and sinus cavities of the models.

### 4.3. Time Measurement of Sinus Filling

The time required to fill up the maxillary sinuses was assessed using video recordings of two models: the right side of the F1 model, representative of a case with a maxillary sinus natural ostium, and the left side of the M1 model after uncinectomy surgery, representing a case with a surgically enlarged maxillary sinus ostium. These tests were conducted using the constant inhalation flow rate that was observed to maximize the aerosol entry into the sinus cavity. The time required for the aerosol droplets to fill the maxillary sinuses during dispensing from the e-vape, as well as the time for the aerosol to escape/deposit in the maxillary sinuses post-dispensing, was measured for both cases. These sinuses were divided into four levels (L0–L3), and the time to reach each level was recorded. Times were measured from the initiation of aerosol dispensing during the dispensing phase and from its cessation during the post-dispensing phase. Due to variability in identifying the boundaries of the aerosol as it fills the maxillary sinuses during aerosol dispensing (or escapes post-dispensing), multiple time measurements were made to assess the time required to reach each of the levels, and the results were averaged.

### 4.4. E-Vape-Generated Aerosol Characterization

Several methods were utilized to assess the characteristics of the aerosols dispensed by the custom-made aerosol dispensing device. A laser diffraction spray particle size analyzer (Spraylink, Dickinson, TX, USA) was used to measure the size distribution of VG-PG aerosols dispensed from the nozzle of the e-vape. Comparative measurements were also made of aerosols dispensed from a mesh nebulizer filled with purified water, as well as from a standard multi-dose nasal spray pump (Hengni, Hengrui Pharmaceuticals, Lianyungang, Jiangsu, China) filled with saline solution (purified water, 0.65% sodium chloride, disodium phosphate, phenylcarbinol, monosodium phosphate, and benzalkonium chloride). Measurements were taken for a set of five separate samples and repeated three times to account for variability during aerosol dispensing.

The mass flow rate of aerosol dispensed from the device was measured as the mass difference of the fog machine fluid tank before and after continuously dispensing aerosol for 15 s. Mass measurements were made using a precision electronic scale (Bonvoisin, San Jose, CA, USA). To address variability, five separate measurements were taken, and the results were averaged.

To examine the plume dynamics, including the plume angle, geometry, and dispersion of the aerosol dispensed from the device, the aerosol plume was captured using a Phantom VEO 1310L high-speed camera (Ametek, Wayne, NJ, USA). Additionally, to assess the behavior of the aerosol after it fills and remains in a chamber (such as the cavity of a paranasal sinus), a test was conducted where VG-PG aerosols were dispensed from the custom aerosol dispensing device to fill a 300 mL beaker. The top of the beaker was then sealed airtight using the palm of a hand, and the behavior of the aerosol trapped in the beaker was recorded and analyzed.

### 4.5. Experimental Model for Assessing E-Vape Aerosol Entry into Sinuses

To understand the mechanisms underlying the aerosol entry into the paranasal sinuses, a simplified dual-bottle setup mimicking the nose-sinus cavity was devised. This setup comprised an empty 500 mL soft drink bottle (representing a nasal cavity passage) connected via a 2 cm length of silicone tube with a 3 mm inner diameter to a 60 mL plastic syringe body (representing a maxillary sinus) that was sealed at both ends. E-vape aerosols were administered at the bottom end of the 500 mL bottle, while a vacuum was connected via a tube to the cap end. The aerosols were generated using the same electronic vaporizer. The flow rate through the large bottle was controlled by a butterfly valve and flow meter to generate the desired flow rates. The objective behind this setup was to validate the effect of the flow rate of the aerosol (administered within the bottle) on aerosol entry and filling of a sinus cavity (represented by the syringe body).

### 4.6. Comparison with Other Nasal Delivery Devices

In this study, the effectiveness of e-vape-generated VG-PG aerosol delivery was compared with several other delivery devices. Each device was tested under the same experimental conditions, including the determined optimal airflow rate and nasal model orientations, to ensure comparability. These tests utilized the M1-Left (post-uncinectomy surgery) and F1-Right models, oriented to mimic an upright head position. The devices considered included a multi-dose nasal spray pump, containing purified water, 0.65% sodium chloride, disodium phosphate, phenylcarbinol, monosodium phosphate, and benzalkonium chloride), a squeeze bottle saline nasal moisturizing spray (Basic Care, Carrington, ND, USA) containing purified water, 0.65% sodium chloride, disodium phosphate, phenylcarbinol, monosodium phosphate, and benzalkonium chloride), a mesh nebulizer, and a humidifier (Pure Enrichment, Huntington Beach, CA, USA). Both the nebulizer and humidifier were filled with purified water. For enhanced visualization of deposition distribution, a fluorescent green dye (GLO Effex, Murrieta, CA, USA) was added to the solutions in the multi-dose nasal spray pump and squeeze bottle, as well as to the purified water in the mesh nebulizer and humidifier. LED lighting in the 385 to 395 nm wavelength range illuminated the dye, making the spray plumes, deposited droplets, and liquid film more visible.

The tests using the multi-dose nasal spray pump and the squeeze bottle involved administering two doses under the same constant unidirectional inhalation airflow rate used in the tests (i.e., 2 L/min). The mist from the humidifier was dispensed via the same tube and nozzle used to administer the aerosol, whereas the fog from the mesh nebulizer was dispensed directly from the silicone tube, which was inserted at one end into the nasal model’s nostril. Mists from both devices were continuously dispensed for more than 1 min. All nozzles were oriented in the models pointing towards the middle meatus, where the ostia to the paranasal sinuses are primarily located.

### 4.7. Statistical Analysis and Numercial Methods

Statistical evaluations were performed using Minitab 21 (State College, PA, USA). The variability in time measurements required for the aerosol to fill the maxillary sinuses was analyzed using a one-way analysis of variance (ANOVA). The time measurement for each experimental scenario was presented as mean ± standard deviation. To further understand the aerosol entry mechanisms, numerical modeling of the airflow in the nose-sinus model was conducted at 2 L/min. More details of the numerical methods can be found in [69].

## 5. Conclusions

This study demonstrates the efficacy of e-vape-generated aerosols in penetrating and filling the paranasal sinuses, particularly the maxillary sinus, under specific administration conditions. The findings highlight the significant potential of e-vape aerosols, composed of a 50/50 mix of vegetable glycerin (VG) and propylene glycol (PG), as a medium for delivering active pharmaceutical ingredients (APIs) to the sinuses. Specific findings are as follows:

1. E-vape-generated VG-PG aerosols effectively enter and deposit in the maxillary sinus by maintaining a positive pressure gradient across the ostial canal.
2. The VG-PG aerosol droplet size, predominantly within the micron range (2.9–3.4 µm), played a crucial role in their effective entry into the sinus.
3. The size of the ostium significantly impacted the entry and retention of e-vape aerosols within the sinus cavities, with larger ostia resulting in faster sinus entry.
4. The sinus filling rate was highest at an inhalation flow rate of 2 L/min and decreased with increasing flow rates, with no entry at 11 L/min and beyond.
5. Gravity and recirculation flow within the sinuses further enhanced the entry and deposition of e-vape aerosols onto the sinus wall.

## Data Availability

The data presented in this study are available on request from the corresponding author.

## Supplementary Materials

The following supporting information can be downloaded at: https://www.mdpi.com/article/10.3390/ph18020142/s1, Video S1: front view of sinus filling in the M1-Left model with post-uncinectomy surgery under unidirectional delivery (with Figure 7b); Video S2: sinus filling in the F1-Right model under unidirectional delivery (with Figure 9); Video S3: sinus filling in the M1-left model with post-uncinectomy surgery under unidirectional delivery (with Figure 10).

## Abbreviations

The following abbreviations are used in this manuscript:

AGI	Active Pharmaceutical Ingredients
MDI	Metered Dose Inhaler
E-Vape	Electronic Vaporizer
VG	Vegetable Glycerin
PG	Propylene Glycol
D50	Median Particle Sizes
FESS	Functional Endoscopic Sinus Surgery
FDA	Food Drug Agency
GRAS	Generally Recognized as Safe
M1	Male Subject 1
F1	Female Subject 1
fps	Frames per Second
NP	Nasopharynx
CFD	Computational Fluid Dynamics
